# Effects of Intravenous or Inhalation Anesthesia on Blood Glucose in Patients with Type 2 Diabetes Mellitus: A PRISMA-Compliant Systematic Review and Meta-Analysis

**DOI:** 10.3390/medicina62010128

**Published:** 2026-01-08

**Authors:** Sang Min Yoon, Hyun Kang, Yoon Ji Choi, Sang Hun Kim, Seongtae Jeong, Sejong Jin

**Affiliations:** 1 Department of Anesthesiology and Pain Medicine, Korea University Ansan Hospital, Ansan 15355, Republic of Korea; mfge00289@korea.ac.kr; 2 Department of Anesthesiology and Pain Medicine, Chung-Ang University College of Medicine, Seoul 06973, Republic of Korea; roman00@naver.com; 3 Department of Anesthesiology and Pain Medicine, Chosun University Hospital, Chosun University School of Medicine, Gwangju 61453, Republic of Korea; ksh3223@chosun.ac.kr; 4 Department of Anesthesiology and Pain Medicine, Chonnam National University Hospital, Chonnam National University Medical School, Gwangju 61469, Republic of Korea; anesjst@jnu.ac.kr; 5 Department of Neuroscience, Korea University College of Medicine, Seoul 02841, Republic of Korea; holicer@korea.ac.kr

**Keywords:** anesthesia, blood glucose, complication, inhalation anesthesia, intravenous anesthesia, type 2 diabetes mellitus

## Abstract

*Background and Objectives*: Perioperative hyperglycemia is associated with increased risks of infection and mortality. Patients with type 2 diabetes mellitus (T2DM) exhibit variable glycemic responses to surgical stress, highlighting the importance of optimal perioperative glucose control. The aim of this study is to conduct a systematic review and meta-analysis comparing the effects of intravenous versus inhalation anesthesia on perioperative blood glucose levels in patients with T2DM undergoing surgery. *Materials and Methods:* We conducted a systematic review and meta-analysis of randomized controlled trials (RCTs) and non-randomized studies identified from Medline, EMBASE, CENTRAL, and Google databases up to 24 October 2024. Eligible studies included adult surgical patients with type 2 diabetes mellitus. Two investigators independently screened studies, extracted data, and assessed methodological quality using the GRADE approach. *Results:* Five studies (3 RCTs and 2 non-RCTs) involving a total of 512 participants were included. Intraoperatively, inhalation anesthesia was associated with significantly higher blood glucose levels compared to intravenous anesthesia (mean difference [MD]: 12.52 mg/dL; 95% confidence interval [CI]: 0.70–24.35) in the overall analysis. However, subgroup analysis by study design showed no significant differences. Postoperatively, inhalation anesthesia resulted in significantly higher glucose levels than intravenous anesthesia, both in the overall analysis (MD: 23.56 mg/dL; 95% CI: 3.65–43.48) and in RCTs alone (MD: 28.20 mg/dL; 95% CI: 3.67–52.73). *Conclusions:* Intravenous anesthesia is associated with lower perioperative blood glucose levels compared to inhalation anesthesia, both during and after surgery. Although the effect was not consistently significant across all subgroups, these findings suggest a potential advantage of intravenous anesthesia in patients with T2DM and warrant validation in larger randomized trials.

## 1. Introduction

Effective perioperative blood glucose management is a critical responsibility of anesthesiologists. Patients with diabetes or preoperative hyperglycemia are at significantly increased risk for perioperative complications and mortality. Notably, the perioperative mortality rate in patients with diabetes is approximately five times higher than that of nondiabetic patients [1,2,3,4,5,6]. Moreover, diabetic patients exhibit a more pronounced glycemic response to surgical stress compared to nondiabetic individuals [7]. As the global prevalence of diabetes increases, the number of diabetic patients undergoing surgery is also rising [8,9]. Given that perioperative hyperglycemia is a modifiable risk factor, optimizing glucose control during the perioperative period is essential for improving surgical outcomes [10].

Surgical stress and anesthetic agents are the primary factors influencing perioperative glucose control. Surgical stress triggers excessive release of proinflammatory cytokines and activates the sympathetic nervous system, leading to increased levels of cortisol, glucagon, catecholamines, and growth hormone. This elevation in counter-regulatory hormones promotes endogenous glucose production and hepatic insulin resistance, while simultaneously reducing glucose uptake by skeletal muscles, ultimately resulting in hyperglycemia [11,12].

Accumulating preclinical and clinical data suggest that volatile anesthetics may disrupt perioperative glucose regulation through impaired insulin secretion and decreased glucose tolerance, potentially predisposing patients to hyperglycemia during the perioperative period [10,13,14,15,16]. Conversely, propofol anesthesia has been reported to attenuate stress-related hormonal responses, improve glycemic control, and maintain more stable hemodynamic parameters, such as blood pressure and heart rate, during surgery in patients with diabetes mellitus [17,18]. Nevertheless, a recent retrospective study of diabetic patients undergoing pulmonary surgery found no significant differences between sevoflurane and propofol anesthesia in terms of perioperative glycemic outcomes, including fasting (à jeun), preoperative, and intraoperative glucose levels, nor in the associated clinical outcomes. [10]. These conflicting results underscore the need for a systematic evaluation of the effects of anesthetic agents on perioperative glucose control in type 2 diabetes mellitus (T2DM).

Although interest in the metabolic effects of anesthetics is increasing, no consensus has been established, and systematic syntheses of the available data remain limited. Therefore, this study aimed to conduct a systematic review and meta-analysis to evaluate the impact of intravenous versus inhalation anesthesia on perioperative blood glucose levels in patients with T2DM undergoing surgery.

## 2. Materials and Methods

This study was conducted in accordance with the Preferred Reporting Items for Systematic Review and Meta-Analysis Protocols (PRISMA-P) and was prospectively registered in the International Prospective Register of Systematic Reviews (PROSPERO; registration number: CRD42024608458). The reporting of this systematic review and meta-analysis followed the PRISMA 2020 guidelines [19].

### 2.1. Search Methods

A comprehensive literature search was performed across Medline (via PubMed), EMBASE, the Cochrane Central Register of Controlled Trials (CENTRAL), and Google Scholar from database inception to 24 October 2024.

The detailed search strategy for PubMed was as follows:

**(“Anesthesia, Intravenous”[MeSH] OR “intravenous anesthesia” OR propofol) AND (“Anesthesia, Inhalation”[MeSH] OR “inhalation anesthesia” OR sevoflurane OR desflurane) AND (“Blood Glucose”[MeSH] OR hyperglycemia OR glycemic control) AND (“Diabetes Mellitus, Type 2”[MeSH] OR type 2 diabetes) AND (“Surgical Procedures, Operative”[MeSH] OR surgery)**

Similar search strategies, adapted to the syntax and indexing terms of each database, were applied for EMBASE, CENTRAL, and Google Scholar. Gray literature was additionally searched using the OpenSIGLE database. No restrictions were imposed on language or publication status. Additional eligible studies were identified by manually reviewing the reference lists of included studies and relevant review articles.

### 2.2. Eligibility Criteria

Studies were included based on the following predefined criteria:

**Population(P)**: Adult patients (aged ≥ 18 years) with a confirmed diagnosis of T2DM undergoing surgical procedures under general anesthesia.

**Intervention(I)**: Administration of general anesthesia using intravenous anesthetic agents, such as propofol.

**Comparison(C)**: Administration of general anesthesia using inhalation anesthetic agents, such as isoflurane, desflurane, and sevoflurane.

**Outcomes(O)**: Quantitative reporting of baseline, intraoperative and/or early postoperative blood glucose levels.

**Study Design(SD)**: Randomized controlled trials (RCTs) and non-randomized comparative studies.

Studies were excluded if they were: (1) animal experiments, (2) narrative or systematic reviews, (3) case reports, or (4) lacked sufficient quantitative data for meta-analysis. Given the limited number of high-quality RCTs in this domain, both RCTs and non-RCTs were included to ensure a comprehensive synthesis of the available evidence.

### 2.3. Selection of Studies and Assessment of Risk of Bias

Two independent investigators initially screened the titles and abstracts of all retrieved records to identify potentially eligible studies. Full-text articles identified by either investigator as potentially relevant were subsequently assessed in detail for final inclusion. Discrepancies in study selection were resolved through discussion; if disagreement persisted, a third reviewer was consulted to reach consensus.

The methodological quality of included studies was independently assessed by two investigators using the Revised Cochrane Risk of Bias tool for randomized trials (RoB 2.0) [20]. The tool evaluates five domains: (1) the randomization process, (2) deviations from intended interventions, (3) missing outcome data, (4) measurement of the outcome, and (5) selection of the reported result. Each domain was rated as having “low risk,” “some concerns,” or “high risk” of bias. The overall risk of bias for each study was determined accordingly.

In addition to risk-of-bias assessment, the overall methodological quality and certainty of evidence were further evaluated by considering study design, sample size, consistency of results, and clinical relevance. Where applicable, the Grading of Recommendations Assessment, Development and Evaluation (GRADE) approach was used to assess the certainty of evidence for key outcomes.

### 2.4. Data Extraction

Two investigators independently extracted relevant data from each included study using a predefined, standardized data collection form. All extracted data were cross-checked for consistency. Discrepancies were resolved through discussion; if consensus could not be reached, a third investigator was consulted.

The data extraction form included the following variables: study title, journal, first author, year of publication, study design, country, language, type of surgery, sample size, anesthesia type, baseline characteristics, risk of bias, intraoperative and postoperative blood glucose levels, and measurement time points.

Data were retrieved from text, tables, or figures, and where necessary, calculated from the reported values. If data were presented only in graphical form, numerical values were extracted using the open-source software Plot Digitizer (version 2.6.8; http://plotdigitizer.sourceforge.net, accessed on 11 November 2024). When essential data were missing or unclear, the corresponding authors were contacted via email to request clarification or additional information.

### 2.5. Statistical Analysis

All statistical analyses were performed using Comprehensive Meta-Analysis software (version 2.0; Englewood, NJ, USA) and Review Manager (RevMan 5.3; The Cochrane Collaboration, Oxford, UK). Two independent reviewers entered the data into the software.

For continuous outcomes, mean differences (MDs) and 95% confidence intervals (CIs) were calculated. Between-study heterogeneity was assessed using Cochran’s Q test, Higgins’ I^2^ statistic, the between-study variance τ^2^ (estimated via the DerSimonian–Laird method), and the prediction interval (PI) [21]. The PI was not calculated when τ^2^ was zero. Random-effects models were used throughout, considering anticipated clinical and methodological heterogeneity across studies [22].

Subgroup analyses were conducted according to study design (RCTs vs. non-RCTs) to explore differences in effect estimates and to investigate potential sources of heterogeneity. Sensitivity analyses were conducted by sequentially omitting individual studies and studies deemed at high risk of bias to evaluate the robustness of the pooled estimates. For studies that reported intraoperative blood glucose at multiple time points, data were pooled across time points to produce a single summary estimate for analysis.

Publication bias was not formally assessed, as the number of included studies was fewer than 10, below the threshold for reliable funnel plot interpretation [23].

### 2.6. Grading of Quality of Evidence

The quality of evidence for each outcome was evaluated using the Grading of Recommendations Assessment, Development, and Evaluation (GRADE) system [24]. Evidence was graded as high, moderate, low, or very low based on five domains: risk of bias, inconsistency, indirectness, imprecision, and publication bias. The GRADE approach was primarily applied to outcomes derived from RCTs. For non-RCT data, results were interpreted with caution due to inherent limitations in study design. Assessment of the certainty of evidence was performed independently by two reviewers.

## 3. Results

### 3.1. Results of the Search

**Figure 1:**
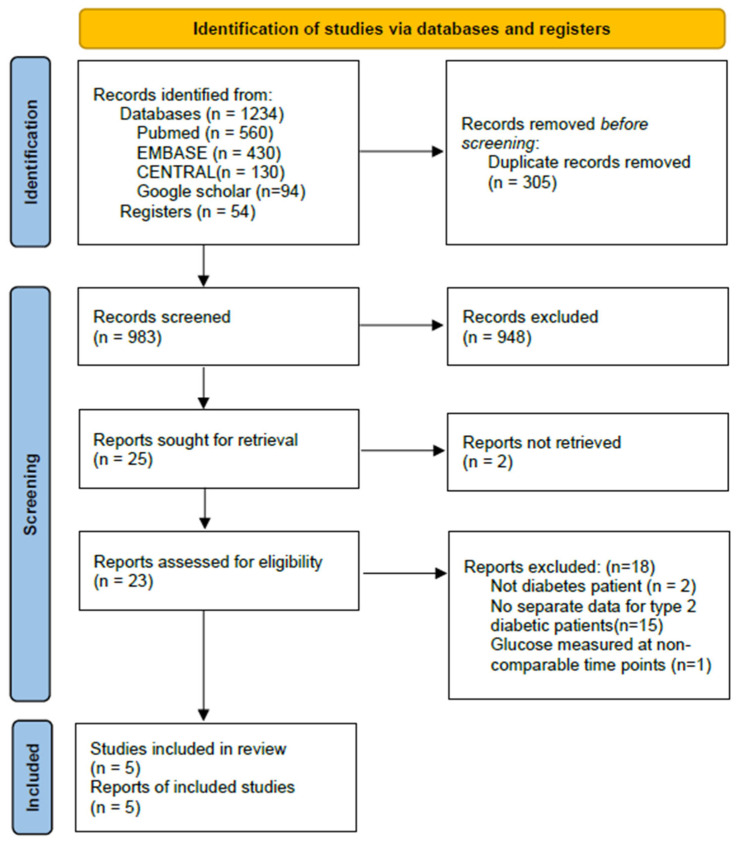
PRISMA 2020 flow diagram of study selection.

A total of 1234 records were identified from databases including 560 from MEDLINE (via PubMed), 430 from EMBASE, 130 from the CENTRAL, 94 from Google scholar and additional 54 records retrieved from Registers. After removing 305 duplicates, 983 records were screened by title and abstract. Of these, 948 were excluded. Twenty-five full-text articles were assessed for eligibility, with 2 excluded because of duplicate titles and 18 excluded for not meeting the inclusion criteria. Finally, 5 studies (3 RCTs and 2 non-RCTs) were included in the meta-analysis. The selection process is illustrated in Figure 1.

### 3.2. Baseline Characteristics of Included Studies

The baseline characteristics of the five included studies are summarized in Table 1. Of these, three were randomized controlled trials (RCTs) and two were non-randomized comparative studies. The types of surgery varied and included gynecologic laparoscopy, abdominal hysterectomy, thoracic surgery, and gastrointestinal procedures. Sample sizes in the intervention arms ranged from 15 to 89 participants. All studies compared intravenous anesthesia using propofol with inhalation anesthesia using sevoflurane, desflurane, or isoflurane. One study [25] used a combination of propofol and remifentanil for intravenous anesthesia, while the others used propofol alone. This pharmacological variation was acknowledged during result interpretation, although it was not assessed in the sensitivity analysis.

### 3.3. Risk of Bias Assessment

**Figure 2:**
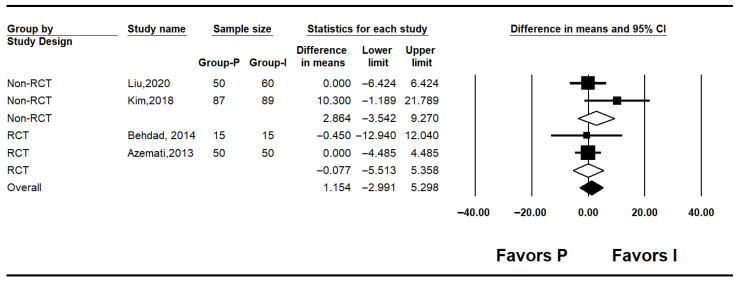
Forest plot showing baseline glucose level [7,10,25,27].

**Figure 3:**
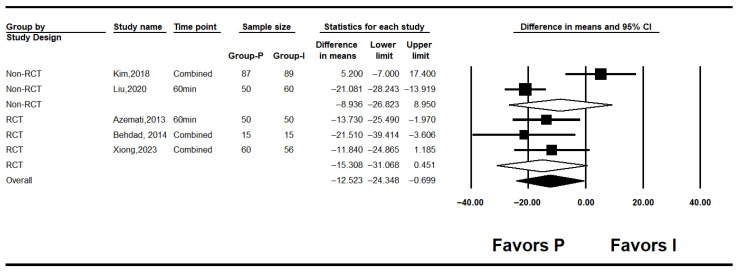
Forest plot showing intraoperative glucose level [7,10,25,26,27].

Risk of bias was assessed using the RoB 2.0 tool for RCTs [7,25,26] and the Risk of Bias Assessment Tool for Nonrandomized Studies (RoBANS) for non-randomized studies [10,27]. The results are summarized in Table 2 and Table 3 and visualized in Figure 2 and Figure 3.

Among the three RCTs, one study [26] was judged to have a low risk of bias across all domains. The remaining two RCTs [7,25] were rated as having “some concerns,” primarily due to insufficient details regarding allocation concealment. However, no serious baseline imbalances were reported. All RCTs showed low risk of bias in the domains related to deviations from intended interventions, outcome measurement, missing outcome data, and selective reporting (Table 2).

For the two non-randomized studies, RoBANS was applied across six domains. One study [10] was rated as low-risk in all domains. The other study [27] was rated as having “unclear” risk in the domain of participant selection due to insufficient reporting of inclusion criteria, while all remaining domains were rated as low-risk (Table 2).

### 3.4. Results of the Meta-Analysis

#### 3.4.1. Baseline Blood Glucose Levels

Four studies reported baseline blood glucose levels [7,10,25,27]. The pooled analysis showed no significant difference between the inhalation anesthesia and propofol groups (mean difference [MD]: 1.154; 95% confidence interval [CI]: −2.991 to 5.298; I^2^ = 0%; P = 0.416; τ^2^ = 0.000). In subgroup analyses by study design, neither the non-RCT (MD: 2.864; 95% CI: −3.542 to 9.270; I^2^ = 57.5%; *p* = 0.125; τ^2^ = 5.522; prediction interval [PI]: −67.30 to 73.03) nor the RCTs (MD: −0.077; 95% CI: −5.513 to 5.358; I^2^ = 0%; *p* = 0.947; τ^2^ = 0.000) demonstrated significant differences (Figure 2).

#### 3.4.2. Intraoperative Blood Glucose Levels

**Figure 4:**
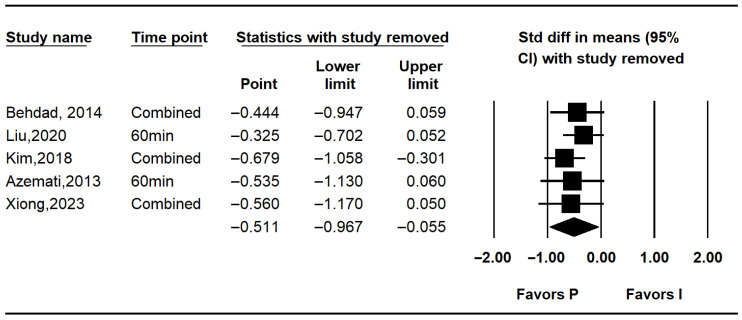
Forest plot showing sensitivity analysis with removing one study at a time for intraoperative glucose level [7,10,25,26,27].

Five studies assessed intraoperative glucose levels [7,10,25,26,27]. Overall, intraoperative glucose was significantly lower in the propofol group compared to the inhalation anesthesia group (MD: −12.523; 95% CI: −24.348 to −0.699; I^2^ = 71.5%; *p* = 0.007; τ^2^ = 9.301; 95% PI: −38.35 to 13.30). Subgroup analysis revealed no significant difference in RCTs (MD: −8.936; 95% CI: −26.823 to 8.950; I^2^ = 92.5%; *p* < 0.001; τ^2^ = 17.863; PI: −235.91 to 218.04), while RCTs showed a non-significant trend favoring propofol (MD: −15.308; 95% CI: −31.068 to 0.451; I^2^ = 0%; *p* = 0.682; τ^2^ = 0.000) (Figure 3). Sensitivity analysis excluding studies by Behdad, Liu, Azemati and Xiong et al. reduced heterogeneity and rendered the overall difference statistically non-significant (Figure 4).

#### 3.4.3. Postoperative Blood Glucose Levels

**Figure 5:**
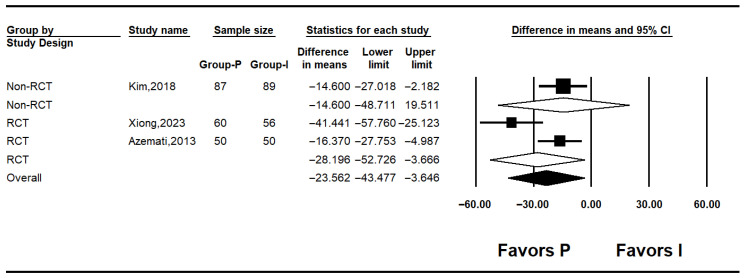
Forest plot showing postoperative glucose level [10,25,26].

**Figure 6:**
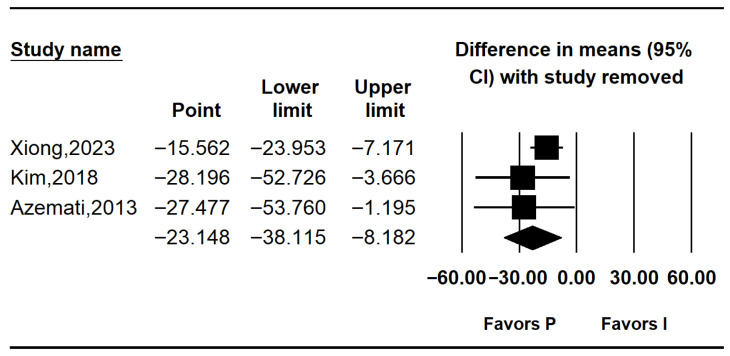
Forest plot showing sensitivity analysis with removing one study at a time for postoperative glucose level [10,25,26].

Three studies reported postoperative glucose levels [10,25,26]. Pooled results indicated significantly lower postoperative glucose in the propofol group (MD: −23.562; 95% CI: −43.477 to −3.646; I^2^ = 74.0%; *p* = 0.021; τ^2^ = 11.336; PI: −72.337 to 25.213). Subgroup analysis showed no significant difference in non-RCTs (MD: −14.600; 95% CI: −48.711 to 19.511; I^2^ = 0%; *p* = 1.000; τ^2^ = 0.000), while RCTs demonstrated a significant reduction in glucose levels with propofol (MD: –28.196; 95% CI: −52.726 to −3.666; I^2^ = 74.0%; *p* = 0.021; τ^2^ = 11.336; PI: −172.23 to115.84) (Figure 5). Sensitivity analysis using the leave-one-out method decreased heterogeneity but did not affect the significance of the results (Figure 6).

### 3.5. Quality of the Evidence

Three outcomes were evaluated using the GRADE system. The quality of the baseline blood glucose level was rated moderate. The quality was rated as low for intraoperative blood glucose level and postoperative blood glucose level (Table 3).

## 4. Discussion

This systematic review and meta-analysis evaluated whether intravenous anesthesia provides superior perioperative glucose control compared to inhalation anesthesia in patients with type 2 diabetes mellitus. Across five studies—including both randomized and non-randomized trials—intravenous anesthesia, primarily using propofol-based regimens, was generally associated with lower intraoperative and postoperative blood glucose levels than inhalation anesthesia. These findings suggest that anesthetic choice may influence glycemic management in diabetic patients undergoing surgery.

No significant difference in baseline glucose levels was observed between groups (MD: 1.154; 95% CI: −2.991 to 5.298; I^2^ = 0%), indicating comparability prior to induction. In contrast, intraoperative glucose levels were significantly lower in the propofol group (MD: −12.523; 95% CI: −24.348 to −0.699), although heterogeneity was substantial (I^2^ = 71.5%). This variability likely reflects differences in study design, types of surgery, anesthetic protocols, patient characteristics, and timing of glucose measurement.

Among the randomized controlled trials, intraoperative glucose levels favored intravenous anesthesia. However, results from non-randomized studies were inconsistent—the study performed by Liu et al. [27]. showed a significant benefit, while the study performed by Kim et al. [10] did not. Sensitivity analyses excluding studies that showed favoring propofol (Behdad et al., Liu et al., Azemati et al., and Xiong et al.) [7,25,26,27] reduced heterogeneity and diminished the statistical significance of the intraoperative finding, suggesting that the overall result may be disproportionately influenced by a subset of studies and should be interpreted cautiously.

Postoperative glucose levels were also significantly lower in the intravenous group (MD: −23.562; 95% CI: −43.477 to −3.646; I^2^ = 74%). Despite heterogeneity, the direction of effect was consistent across all studies. Leave-one-out sensitivity analysis confirmed the robustness of this finding, as the pooled effect remained significant regardless of which study was excluded.

Our findings are consistent with previous reports indicating that intravenous anesthesia, particularly propofol, is associated with better perioperative glycemic control compared to inhalation agents [17,28,29]. Inhalational anesthetics such as sevoflurane and isoflurane have been shown to impair glucose utilization, increase insulin resistance, and attenuate compensatory responses to hyperglycemia—even in the absence of surgical stress[30,31,32].

Surgical stress activates sympathetic nervous system and the hypothalamic–pituitary–adrenal (HPA) axis, leading to elevated levels of cortisol, catecholamines, glucagon, and growth hormone, which disrupt glycemic regulation [5,33]. Propofol is known to attenuate the activation of the HPA axis and suppress the release of stress hormones such as cortisol and catecholamines [29]. This blunted neuroendocrine response may contribute to reduced gluconeogenesis and glycogenolysis during surgical stress. Furthermore, propofol appears to exert less inhibitory effect on pancreatic insulin secretion compared to inhalational agents and may even enhance peripheral glucose uptake through improved insulin signaling [34]. In contrast, inhalational anesthetics such as sevoflurane and isoflurane have been associated with impaired insulin secretion and increased insulin resistance [30,35]. It has been shown that these agents reverse glucose-induced inhibition of ATP-sensitive potassium channels in pancreatic β-cells, thereby suppressing insulin release and contributing to perioperative hyperglycemia [13,30]. These pharmacologic differences may account for the observed differences in glucose control in our study.

Perioperative hyperglycemia is a known risk factor for adverse surgical outcomes, including infections, delayed wound healing, cardiovascular complications, and prolonged hospital stay—particularly in diabetic patients [36,37]. Thus, anesthetic techniques that support glycemic stability may contribute to improved outcomes. Although anesthetic selection is typically based on surgical and institutional factors, our findings suggest that glycemic effects should also be considered when managing patients with diabetes.

This study has several strengths. To our knowledge, it is the first meta-analysis specifically comparing the effects of intravenous versus inhalation anesthesia on perioperative glucose levels in type 2 diabetic patients. By including both randomized and non-randomized studies, we were able to assess a broader range of clinical settings. Subgroup and sensitivity analyses improved the reliability of findings, while risk of bias and quality of evidence were rigorously evaluated using established tools.

Nevertheless, limitations exist. The total number of studies was only five, with a total of 512 participants, and sample sizes were limited, reducing statistical power, making results fragile to single-study effects, and preventing firm clinical conclusions. High heterogeneity, especially in non-randomized studies, may reflect clinical and methodological differences. This suggests that studies measured different effect sizes, and that other factors besides anesthetics may also influence glycemic outcomes. All included studies used propofol as the intravenous agent, limiting generalizability to other intravenous anesthetics. Additionally, although some studies reported biomarkers such as interleukin-1β or cortisol, this meta-analysis focused solely on glucose levels, precluding mechanistic insights into endocrine or immune modulation. Future large-scale randomized trials are needed to confirm these findings and clarify underlying mechanisms. Incorporating biomarkers of inflammation, stress response, and insulin sensitivity would help elucidate how anesthetic techniques influence perioperative glucose metabolism in diabetic patients.

## 5. Conclusions

In conclusion, intravenous anesthesia is associated with lower perioperative blood glucose levels compared to inhalation anesthesia, both during and after surgery. Although the effect was not consistently significant across all subgroups, these findings suggest a potential advantage of intravenous anesthesia in patients with T2DM and warrant validation in larger randomized trials.

## Figures and Tables

**Table 1:** Baseline characteristics of included studies.

Author and Year	Type of Surgery	Study Design	Intervention Group		Control Group	
			Type of Anesthesia	Sample Size (n)	Type of Anesthesia	Sample Size (n)
Azemati, 2013 [25]	Gynecologic laparoscopy	RCT	Propofol + remifentanil	50	Isoflurane + remifentanil	50
Behdad, 2014 [7]	Abdominal hysterectomy	RCT	Propofol	15	Sevoflurane	15
Xiong, 2023 [26]	General	RCT	Propofol	56	Desflurane	60
Kim, 2018 [10]	Thoracic	Non-RCT	Propofol	89	Sevoflurane	87
Liu, 2020 [27]	Gastrointestinal	Non-RCT	Propofol	60	Sevoflurane	50

n: number, RCT: randomized controlled trial.

**Table 2:** Quality assessment of included studies.

Risk of Bias Assessment of Randomized Controlled Trials Using RoB 2.0						
Study	Domain					
	Randomization process	Deviations from intended interventions	Missing outcome data	Measurement of the outcome	Selection of the reported result	Overall Bias
Azemati, 2013 [25]	Some concerns *	Low risk	Low risk	Low risk	Low risk	Some concerns
Behdad, 2014 [7]	Some concerns *	Low risk	Low risk	Low risk	Low risk	Some concerns
Xiong, 2023 [26]	Low risk	Low risk	Low risk	Low risk	Low risk	Low risk
**Risk of Bias Assessment of Non-Randomized Studies Using RoBANS**						
**Study**	**Domain**					
	**Selection of participants**	**Confounding variables**	**Intervention measurement**	**Blinding of outcome assessment**	**Incomplete outcome data**	**Selective outcome reporting**
Kim, 2018 [10]	Low risk	Low risk	Low risk	Low risk	Low risk	Low risk
Liu, 2020 [27]	Unclear	Low risk	Low risk	Low risk	Low risk	Low risk

RoB 2.0; Revised Cochrane risk of bias tool for randomized trials, RoBANS, Risk of Bias Assessment Tool for Nonrandomized Studies. * Downgraded due to the absence of information regarding allocation concealment, although the lack of baseline imbalance suggests no major concerns with the randomization process.

**Table 3:** The GRADE evidence quality for each outcome.

	No of Studies	No of Patients	Quality Assessment					Quality
			ROB	Inconsistency	Indirectness	Imprecision	Publication Bias	
Baseline Blood Glucose Levels	4	416	Not serious	Not serious	Not serious	Serious ^†^	NA	⨁⨁⨁◯ Moderate
Intraoperative Blood Glucose Levels	5	532	Not serious	Serious *	Not serious	Serious ^†^	NA	⨁⨁◯◯ Low
Postoperative Blood Glucose Levels	3	392	Not serious	Serious *	Not serious	Serious ^†^	NA	⨁⨁◯◯ Low

* We downgraded the certainty of evidence by one level for inconsistency due to heterogeneity among studies, with an I^2^ statistic over 50% or P_chi_^2^ less than 0.05. ^†^ We downgraded imprecision by one level for serious imprecision due to very wide confidence intervals, including both substantial harms and benefits, and/or predictive interval includes 0.

## Data Availability

The data presented in this study is available on request from the corresponding author. Any requests will be reviewed against compliance with ethical, scientific, regulatory, and legal requirements. Requests to access the datasets should be directed to roman00@naver.com.

## Abbreviations

The following abbreviations are used in this manuscript:

PM2.5	Particulate matter
NHI	National Health Insurance
ORs	Odds ratios
CIs	Confidence intervals
